# Dietary intake patterns and nutritional status of women of reproductive age in Nepal: findings from a health survey

**DOI:** 10.1186/s13690-016-0114-3

**Published:** 2016-01-28

**Authors:** Shiva Bhandari, Jamuna Tamrakar Sayami, Pukar Thapa, Matina Sayami, Bishnu Prasad Kandel, Megha Raj Banjara

**Affiliations:** Multivitamin-mineral Supplementation Project, Health Resources Consultancy Pvt. Ltd., Kuleshwor Kathmandu, Nepal; Public Health and Infectious Disease Research Center (PHIDReC), New Baneshwor Kathmandu, Nepal; National Center for Health Professions Education, Institute of Medicine, Tribhuvan University, Maharajgunj Kathmandu, Nepal; Department of Internal Medicine, Lumbini Medical College, Kathmandu University, Tansen, Palpa Nepal; Department of Internal Medicine, Maharajgunj Medical College, Institute of Medicine, Tribhuvan University, Maharajgunj Kathmandu, Nepal; Department of Surgery, Institute of Medicine, Tribhuvan University, Maharajgunj Kathmandu, Nepal; Central Department of Microbiology, Tribhuvan University, Kirtipur Kathmandu, Nepal

**Keywords:** Anemia, Body Mass Index, Dietary intake pattern, Nepal, Nutritional status, Reproductive age

## Abstract

**Background:**

Improper dietary intake pattern in women of reproductive age in Nepal has resulted in the deficiency of essential nutrients. Adequate nutritional status and proper dietary intake pattern of women improves maternal and child health. The objective of this study was to assess the nutritional status and dietary intake pattern among the women and associated factors.

**Methods:**

Data collection at households and health check-up camps were conducted in selected Village Development Committees of nine districts in three ecological regions (Mountain, Hill and Terai) of Nepal from September 2011 to August 2012. Women of reproductive age (15 to 49 years) were the study subjects. At the household interview, structured questionnaires were used to obtain information on socio-demographic characteristics, anthropometric measurements, dietary intake pattern, consumption of junk foods, animal rearing, agricultural products, possession of kitchen garden, pregnancy status and anemia. Dietary intake pattern was determined by information collected through the structured questionnaires comprising of food items-cereals, pulses/legumes, vegetables, meat, fruits and milk and milk products. Health check-up camps were conducted in the local health facilities where qualified doctors, nurses and laboratory technicians performed physical examination of the women, confirmed their pregnancy and conducted hematocrit tests. The data was entered and analyzed using SPSS.

**Results:**

Altogether 21,111 women were interviewed. More than a quarter of the women in Terai were malnourished as indicated by low body mass index (BMI < 18.5 Kg/m^2^). Among the dietary intake pattern, the majority of women consumed cereals at least once a day in all three ecological regions. The majority of women in Mountain consumed pulses/legumes thrice a week. In Terai, the majority of women consumed vegetables thrice a week. In all three ecological regions, the majority of women consumed meat and meat products and fruits once a week. About thirty percent of women consumed milk and milk products once a day in all three ecological regions. The non-use of iodized salt by Terai women was the highest (5.3 %, *n* = 303). In all the ecological regions, cereals and vegetables were produced in the majority of the participants’ households in comparison of fruits, poultry and goat/sheep. The women of age 15 to 24 years were 2.7 times more likely to be malnourished than women of 35 to 49 years age (aOR = 2.7, CI = 2.5,3.0). The unemployed women had nearly two times more chances of being malnourished than women doing manual work (aOR = 1.9, 95 % CI = 1.5,2.2). In Terai, women were five times more likely to be malnourished (aOR = 0.2, CI = 0.1,0.2) and 20 times more likely to be anemic (aOR = 0.05, CI = 0.04,0.07) than women in Mountain. The pregnant women were five times more likely to be anemic than non-pregnant women (aOR = 0.2, CI = 0.2,0.3).

**Conclusions:**

The nutritional status of women of reproductive age is still poor especially in Terai and the dietary intake pattern is not adequate. It suggests improving nutritional status and feeding habits especially intake of meat, fruits and vegetables focusing on reproductive aged women.

## Background

Dietary intake pattern plays a significant role in human health [1, 2]. Improper and inadequate dietary intake pattern especially in women of reproductive age have resulted in the deficiency of essential nutrients especially during pregnancy and lactation in Nepal, where 18 % of women are malnourished and 35 % are anemic [3], which pose threat to physical, mental and social well being of women [4]. In addition, reproductive biology, poverty, lack of education, socio-cultural traditions and disparities in household contribute to under nutrition in women [5]. Those women who consume limited animal source foods, fruits and vegetables, increase their risk of micronutrient deficiencies [6]. Women on low protein and carbohydrate diets can be severely malnourished mothers and are at increased risk of child mortality [7].

Nutritional status is an indication of the overall well being of a population. Adequate nutritional status of women is important for good health and increased work capacity of women themselves as well as for the health of their offspring [4]. Poor nutrition is indicative of greater health risk to both mother and children born to them [8]. The health risk it could pose for women necessitates continuous monitoring of their nutritional status and dietary intake especially in poor resource countries like Nepal. The current literatures provide limited information regarding dietary intake pattern and nutritional status in Nepal. The objective of this study was, therefore, to assess the dietary intake pattern and nutritional status of women of reproductive age and associated factors in Nepal. In other words, the study intends to measure diversity in dietary behavior with regard to important and commonly consumed foods groups and nutritional status of women but not how much food do individuals consume or the average calorie intake.

## Methods

The data presented in the study are part of an intervention study and are the pre-intervention measurement, where women of reproductive age were supplemented with multivitamin-minerals and assesses for the perinatal outcome. Therefore, the sampling of Village Development Committees (VDCs) is not random but targeted.

### Study area

Nepal is divided into three distinct ecological regions: Mountain, Hill and Terai. Mountain (occupies 35 % of the total land area) has rocky terrain and lies to the north, the middle is Hill (occupies 42 % of the total land area) and Terai (occupies 23 % of the total land area) lies to the southern part of the country and has relatively flat terrain. According to national population and housing census, 2011, Mountain has only about 6.7 % (1,781,792) of the total population and 42.3 % of who lives under poverty; Hill has 43 % (11,394,007) of the total population, 24.3 % of who lives under poverty; Terai has 50.3 % (13,318,705) of the population lives here and 23.4 % are under poverty [9]. Similarly, the female literacy is 46.7, 33.3 and 44.9 % in Mountain, Hill and Terai regions respectively [9]. Each region is subdivided into districts (a total of 75 districts in Nepal) and within the districts are Village Development Committees (VDCs). For this study, nine districts (Dolakha-Mountain; Illam, Kavrepalanchowk, Kathmandu, Lamjung and Kaski-Hill; Sarlahi, Nawalparasi and Kailali-Terai) were selected. Within nine districts, three to eight VDCs were selected. The number of selected VDCs in the study districts was three in Dolakha and Nawalparasi, four in Lamjung, six in Illam, Kavrepalanchowk, Sarlahi and Kailali, eight in Kaski and Kathmandu.

### Study participants, design and health camps

The study population was women of reproductive age (15–49 years) residing in the selected VDCs. A household level study was conducted from September 2011to August 2012 in the selected study areas. Interview of reproductive age women was done at the household using structured questionnaires. Information regarding socio-demographic characteristics, anthropometric measurements, dietary intake pattern, animal rearing, agricultural products, possession of kitchen garden, use of iodized salt, nutrition education by health institutions, pregnancy status and anemia were obtained. A non-random sampling technique was used for the selection of districts and VDCs. The districts covering all three ecological regions were selected consulting Ministry of Health and Population, Nepal and the VDCs were selected in consultation with District Public/Health Offices. It was assumed that those areas were supposed to have poor health and nutrition. Interview of study subjects was started from one end of the VDC by selecting a house. All the women of reproductive age living in the house were interviewed. However, if there were not any study subjects in the house, or they were out of the house at the time of data collection, the house was skipped and nearby house was selected. 12.5 % of the study participants could not be included due to their absence. Local health facility staffs and female community health volunteers (FCHVs) helped to find the location of houses. First, the questionnaires were made in English. Later, they were translated into Nepali so that the participant women could easily understand.

All the women interviewed were invited to attend health check-up camp through field supervisor at the time of interview. In health check-up camps participants are informed to come to a particular place (generally nearby health facility) and doctors, nurses, laboratory technicians (whenever required) and other health personnel check the general or specific health condition of the participants. The health camps were conducted in the health facilities of the concerned VDCs for clinical assessment. A qualified doctor conducted physical examination of women and pregnancy status. Nurse and health facility staffs were involved in anthropometric measurements of the women. To identify anemia in women, capillary blood samples were tested for hematocrit determination by laboratory technician with hematocrit machine (Heamata STAT-II, STI Separation Technology Inc., USA). A structured nutrition education program was also conducted at end of the health check-up highlighting the importance of proper nutrition for reproductive aged women to make them aware about nutrition.

### Sample size and design

Since the present study is a part of baseline survey of an interventional study (supplementation of multivitamin-minerals and assessment of perinatal outcome), total sample in this study was obtained on the basis of sample size calculated for that study. In addition, sampling design was employed for that interventional study. A total of 21,371 women participated in the survey. However, only 21,111 participants were included in the present study for analysis because of the exclusion of missing values and incomplete information (260 participants).

### Data collection procedures

In each household, separate confidential interview of the women was conducted in a convenient place. About half an hour was taken for the whole procedure of conducting questionnaires. Before introducing questionnaires, the women were informed about the purpose of the study. They were made aware of the fact that they can withdraw from the study at any stage of the study. Written consent was taken from each study subject. For women who were below 18 years of age, written consent from them as well from their parents/guardians was obtained.

### Dietary intake data collection

Dietary intake pattern was determined by using a tool previously used in India [10]. This tool was modified with consultation with experts to include as much information as possible. The data was collected through the questionnaires that comprised of six groups of food items: (i) cereals (ii) pulses/legumes (iii) vegetables (iv) meat (v) fruits and (vi) milk and milk products. In addition, junk foods (sweetened beverages, instant noodles, cookies and biscuits available in local markets) were included in different category. Cereals included rice, wheat, millet, maize and barley; pulses/legumes comprised of beans, peas, soybeans, grams and others used to make lentils; vegetables included green leafy vegetables, cauliflower, ladies finger, brinjal, pumpkin, and others whatever they grew in their fields or buy from market to make curry; meat included chicken, mutton, fish, buff, pork and beef; fruits comprised of seasonal fruits grown in their fields or seasonal/non-seasonal fruits bought from markets; milk and its products consisted of milk, curd and cheese. The participating women were asked ‘how often do you yourself consume the food groups: daily, thrice a week, weekly, monthly or never?’ The interviewers asked about the group of food not the individual items. If the participants did not understand, examples of food items were provided and they were probed regarding the consumption of specific food items. Among these foods, cereals are rich in carbohydrates, pulses/legumes, meat and milk products are rich in protein, vegetables are rich source of iron, folic acid, vitamin A, carotene, riboflavin and calcium, whereas fruits contain especially vitamin C, vitamin A and minerals. It should be noted that the survey did not provide any information related to the quantity or level of food consumption over time.

### Ethical approval

Ethical approval was obtained from Ethical Committee of the Nepal Health Research Council (NHRC Reg. No. 5/2011) as per national health research policy. Similarly, written approvals from respected District Public/Health Offices were taken.

### Anthropometric measurement and anemia

Weights of the women were measured to the nearest 0.1 kg on a battery powered digital scale (Seca GmBH & Co.kg., Germany) and heights were measured to the nearest centimeter using a height scale following standard anthropometric techniques [11]. For weight and height measurements, study subjects removed their shoes, removed their jackets and wore light clothing. Body mass index (BMI) of the study subjects was calculated by dividing the weight in kilogram to the height in meter squared (Kg/m^2^). BMI less than 18.5 was considered as underweight (malnourished) [12] and anemia was defined as a hematocrit value less than 35 % and normal as more than 35 % [13].

### Data entry and analysis

Data entry and analysis was performed by using SPSS for windows version 11.5 (SPSS Inc., Chicago). The obtained data was weighted for districts assuming that all women within a district have similar pattern of dietary intake. Descriptive analysis was done and the result was expressed in percentage. Inferential statistics was calculated using chi-square test. Adjusted odds ratio (aOR) after adjusting for educational status, age (in years), employment, ethnicity, ecological regions, pregnancy and nutrition education by any organizations was calculated. Binary logistic regression with 95 % confidence interval (CI) was performed and p-value less than 0.05 was considered significant.

## Results

In total, information from 21,111 women interviewed were analyzed. The mean height (±SD) of women was 151.5 (±6.1) cm and majority of women, 17.8 % (*n* = 162), of height less than or equal to 145 cm (stunted) live in Mountain. Similarly, the mean weight (±SD) of the women was 49.3 (±8.2) Kg and nearly half of the women in Terai had weight less than or equal to 45 Kg. The mean BMI (±SD) of the women was 21.5 (±3.5) Kg/m^2^ and more than a quarter of the women in Terai had BMI below less than 18.5 Kg/m^2^ (malnourished) (Table 1).

**Table 1:** Anthropometric status of women of reproductive age, Nepal, 2012

Variables	Mountain (%^a^)	Hill (%^a^)	Terai (%^a^)
Height (in cm) (*n* = 14,366)			
≤145 cm	161 (17.8)	1443 (16.4)	733 (15.0)
> 145 cm	745 (82.2)	6883 (83.6)	4256 (85.0)
Mean ± SD (151.5 ± 6.1)	151.3 ± 6.4	151.0 ± 6.1	152.2 ± 6.0
Weight (in kg) (*n* = 13,369)^b^			
≤ 45 kg	195 (21.6)	2638 (33.1)	2184 (48.8)
> 45 kg	693 (78.4)	5193 (66.9)	2466 (51.2)
Mean ± SD (49.3 ± 8.2)	52.4 ± 7.9	50.1 ± 8.5	47.3 ± 7.5
Body Mass Index (BMI, Kg/m^2^) (*n* = 13,369)^b^			
< 18.5	54 (6.0)	1161 (15.4)	1235 (27.9)
18.5 to 24.9	617 (69.2)	5262 (65.9)	3083 (65.8)
≥ 25	217 (24.8)	1408 (18.7)	332 (6.3)
Mean ± SD (21.5 ± 3.5)	22.9 ± 3.4	22.0 ± 3.6	20.4 ± 3.0

^a^Weighted percent and means

^b^Pregnant women were excluded

The majority of women in all regions possessed land for kitchen garden. More than half of the women in Mountain did not have any health institutions providing awareness on nutrition. In Terai, still 19.4 % (*n* = 1329) of women had not heard about iodine, the essential micronutrient, while 5.3 % (*n* = 303) of women in their household did not use iodized salt with two children logo. Almost eighty-five percent of the women consumed junk foods in Mountain and Hill while about two-third of women consumed such foods in Terai (Table 2).

**Table 2:** Variables associated with dietary intake practice of women of reproductive age, Nepal, 2012 (*N* = 21,111)

Variables	Mountain (*n* = 2320) (%^a^)	Hill (*n* = 12,372) (%^a^)	Terai (*n* = 6419) (%^a^)
Possession of land for kitchen garden			
Yes	2148 (92.6)	10914 (85.4)	5345 (83.3)
No	172 (7.4)	1458 (14.6)	1074 (16.7)
Health institutions providing awareness on nutrition			
Yes	1073 (46.3)	7516 (58.6)	3622 (57.5)
No	1247 (53.7)	4856 (41.4)	2797 (42.5)
Women heard of iodine			
Yes	1958 (84.4)	10903 (88.0)	5090 (80.6)
No	362 (15.6)	1469 (12.0)	1329 (19.4)
Use of iodized salt with two children logo			
Yes	2189 (94.4)	11777 (93.9)	6116 (94.4)
No	131 (5.6)	595 (6.1)	303 (5.6)
Consumption of junk foods			
Yes	1955 (84.3)	10403 (85.7)	4778 (75.6)
No	365 (15.7)	1969 (14.3)	1641 (24.4)

^a^Weighted percent

**Fig. 1:**
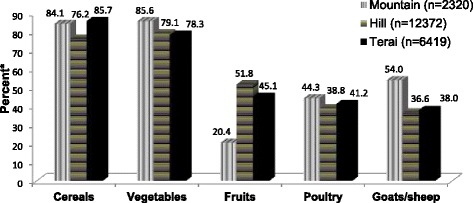
Agricultural production from households of women of reproductive age, Nepal, 2012 (*N* = 21,111). *weighted by districts

In Terai, 85.7 % (*n* = 5484) women in their households produced cereals and 78.3 % (*n* = 4953) women produced vegetables. The majority of women (85.6 %, *n* = 1985) in Mountain and 79.1 % (*n* = 10044) in Hill produced vegetables in their households. Women’s household production of fruits was the least in Mountain while production of meat and meat products was the least in Hill (Fig. 1).

Regarding the frequency of consumption of staple foods rich in carbohydrates, more than eighty-seven percent of the women in Hill and Terai consumed cereals more than once a day and in Mountain more than two-third of the women consumed cereals more than once a day. The majority of women in Hill and Terai consumed pulses/legumes once a day while the majority of women in Mountain consumed pulses/legumes thrice a week (Table 3).

**Table 3:** Frequency of consumption of carbohydrate and protein rich foods by women of reproductive age, Nepal, 2012 (*N* = 21,111)

Food and frequency	Mountain (*n* = 2334) (%^a^)	Hill (*n* = 12,543) (%^a^)	Terai (*n* = 6494) (%^a^)
Cereals			
More than once a day	1746 (75.3)	10815 (87.7)	5556 (87.3)
Once a day	359 (15.5)	1035 (7.7)	441 (6.3)
Thrice a week	109 (4.7)	216 (1.8)	261 (4.0)
Once a week	94 (4.1)	134 (1.1)	122 (1.8)
Once a month	12 (0.5)	166 (1.7)	31 (0.4)
Never	0 (0.0)	6 (0.1)	8 (0.1)
Pulses/Legumes			
More than once a day	204 (8.8)	3709 (32.0)	1305 (20.3)
Once a day	632 (27.2)	4864 (35.5)	2422 (39.2)
Thrice a week	682 (29.4)	2462 (21.1)	1729 (26.3)
Once a week	571 (24.6)	1048 (9.3)	676 (10.0)
Once a month	223 (9.6)	258 (1.8)	249 (3.7)
Never	8 (0.3)	31 (0.2)	38 (0.6)

^a^Weighted percent

In Mountain, more than forty-five percent of women consumed green vegetables once and more than once a day. However, in Terai, the majority of women consumed vegetables thrice a week. In all three ecological regions, the majority of women consumed meat and fruits once a week. About thirty percent of women consumed milk and milk products once a day in all three ecological regions (Table 4).

**Table 4:** Frequency of consumption of micro-nutrients rich foods by women of reproductive age, Nepal, 2012 (*N* = 21,111)

Food and frequency	Mountain (*n* = 2334) (%^a^)	Hill (*n* = 12,543) (%^a^)	Terai (*n* = 6494) (%^a^)
Vegetables			
More than once a day	1049 (45.2)	3499 (29.1)	906 (12.6)
Once a day	1054 (45.4)	5516 (39.8)	2087 (32.2)
Thrice a week	168 (7.2)	2514 (23.0)	2295 (37.0)
Once a week	40 (1.7)	757 (7.2)	914 (14.8 )
Once a month	9 (0.4)	70 (0.7)	195 (3.1)
Never	0 (0.0)	16 (0.1)	22 (0.3)
Meat			
More than once a day	14 (0.6)	178 (1.4)	139 (2.0)
Once a day	68 (2.9)	510 (4.5)	207 (3.0)
Thrice a week	390 (16.8)	2802 (26.0)	1211 (17.6)
Once a week	1183 (51.0)	5805 (48.6)	3213 (50.7)
Once a month	632 (27.2)	2258 (12.1)	1463 (23.8)
Never	33 (1.4)	819 (7.4)	186 (2.9)
Fruits			
More than once a day	20 (0.9)	699 (5.6)	349 (5.1)
Once a day	92 (4.0)	2190 (16.0)	636 (9.4)
Thrice a week	217 (9.4)	2618 (21.3)	1513 (24.4)
Once a week	1116 (48.1)	4449 (36.9)	2467 (39.6)
Once a month	865 (37.3)	2265 (18.8)	1362 (20.3)
Never	10 (0.4)	151 (1.4)	92 (1.3)
Milk and milk products			
More than once a day	278 (12.0)	1827 (15.9)	457 (6.3)
Once a day	632 (27.2)	4047 (31.1)	1773 (27.8)
Thrice a week	294 (12.7)	2001 (16.3)	1440 (23.6)
Once a week	401 (17.3)	1471 (14.2)	941 (14.4)
Once a month	416 (17.9)	1977 (13.6)	973 (14.1)
Never	399 (12.9)	1049 (8.9)	835 (13.8)

^a^Weighted percent

Surprisingly, the women having formal education were 1.4 times more likely to be malnourished than the women having informal education (aOR = 0.7, 95 % CI = 0.6,0.8). The women of age 15 to 24 years were about three times more likely to be malnourished than women of 35 to 49 years age (aOR = 2.7, 95 % CI = 2.5,3.0). The unemployed women had nearly two times more chances of being malnourished than women doing manual work (aOR = 1.9, 95 % CI = 1.5,2.2). Women of upper caste were 2.5 times more likely to be malnourished than the women of religious minorities (aOR = 0.4, 95 % CI = 0.3,0.5). The women of Terai were five times more likely to be malnourished than the women of Mountain (aOR = 0.2, 95 % CI = 0.1,0.2). The women who did not get nutrition education from any organizations were 1.1 times more likely to be malnourished than those women who got (aOR = 1.1, 95 % CI = 1.0,1.2) (Table 5).

**Table 5:** Association of socio-demographic variables with BMI of women of reproductive age, Nepal, 2012 (*n* = 13,369)

Variables	Malnourished (%^a^)	Normal (%^a^)	aOR^bc^ (95 % CI)
Educational Status			
Illiterate	561 (18.7)	2594 (81.3)	1.0 (0.9,1.1)
Informal education	216 (12.2)	1732 (87.8)	0.7 (0.6,0.8)**
Formal education	1673 (20.9)	6593 (79.1)	1.0
Age (in years)			
15–24	1560 (26.0)	4649(74.0)	2.7 (2.5,3.0)**
25–34	578 (13.6)	3584 (86.4)	1.3 (1.2,1.4)**
35–49	312 (10.9)	2686 (89.1)	1.0
Employment			
Unemployed	1036 (23.5)	3303 (76.5)	1.9 (1.5,2.2)**
Formal employment	48 (7.9)	583 (92.1)	0.8 (0.6,1.0)
Agriculture	1345 (17.7)	6879 (82.3)	1.6 (1.3,1.9)**
Manual work	21 (10.1)	154 (89.9)	1.0
Ethnicity			
Dalit	248 (19.1)	1090 (80.9)	0.8 (0.7,0.8)**
Disadvantaged janajati	825 (18.1)	4184 (81.9)	0.6 (0.5,0.6)**
Disadvantaged non-dalit Terai caste	258 (23.8)	800 (76.2)	0.6 (0.5,0.6)**
Religious minorities	18 (19.1)	63 (80.9)	0.4 (0.3,0.5)**
Relatively advantaged janajati	128 (12.0)	1047 (88.0)	0.6 (0.5,0.6)**
Upper caste	973 (21.5)	3735 (78.5)	1.0
Ecological regions			
Mountain	54 (6.0)	834 (94.0)	0.2 (0.1,0.2)**
Hill	1161 (15.4)	6670 (84.6)	0.4 (0.4,0.5)**
Terai	1235 (27.9)	3415 (72.1)	1.0
Nutrition education by any organizations			
Yes	1369 (18.6)	6393 (81.4)	1.0
No	1081(20.3)	4526 (79.7)	1.1 (1.0,1.2)**

^a^Weighted percent

aOR^b^ weighted adjusted odds ratio, 1 = reference

^c^The odds ratio are adjusted for all variables

**P < 0.001

Janajati includes all the castes that fall under marginalized group except dalit (lower caste)

The women having formal education were 1.4 times more likely to be anemic than the women having informal education (aOR = 0.7, 95 % CI = 0.6,0.8) and illiterate women (aOR = 0.7, 95 % CI = 0.7,0.8). The women of age 15 to 24 years were 1.3 times more likely to be anemic than the women of 35 to 49 years (aOR = 1.3, 95 % CI = 1.0,1.3). The women employed in agriculture had nearly two times more chances of being anemic than women doing manual work (aOR = 1.7, 95 % CI = 1.5,2.0). The women of upper caste were two times more likely to be anemic than disadvantaged non-dalit Terai caste women (aOR = 0.5, 95 % CI = 0.5,0.6) and religious minorities women (aOR = 0.5, 95 % CI = 0.4,0.6). Pregnancy was greatly associated with anemia as the pregnant women were five times more likely to be anemic than non-pregnant women (aOR = 0.2, 95 % CI = 0.2,0.3). The women of Terai were 20 times more likely to be anemic than the women of Mountain (aOR = 0.05, 95 % CI = 0.04,0.07) (Table 6).

**Table 6:** Association of socio-demographic variables with anemia of women of reproductive age, Nepal, 2012 (*n* = 14,222)

Variables	Anemic (%^a^)	Normal (%^a^)	aOR^bc^ (95 % CI)
Educational Status			
Illiterate	613 (21.3)	2720 (78.7)	0.7 (0.7,0.8)**
Informal education	382 (22.5)	1652 (77.5)	0.7 (0.6,0.8)**
Formal education	2422 (31.4)	6433 (68.6)	1.0
Age (in years)			
15–24	1809 (31.3)	4945 (68.7)	1.3 (1.0,1.3)**
25–34	1048 (26.8)	3386 (73.2)	1.1 (1.0,1.2)**
35–49	560 (22.1)	2474 (77.9)	1.0
Employment			
Unemployed	1272 (29.8)	3369 (70.2)	1.6 (1.4,1.8)**
Formal employment	149 (25.4)	523 (74.6)	1.4 (1.2,1.7)**
Agriculture	1957 (27.7)	6755 (72.3)	1.7 (1.5,2.0)**
Manual work	39 (20.0)	158 (80.0)	1.0
Ethnicity			
Dalit	363 (28.2)	1083 (71.8)	0.8 (0.7,0.9)**
Disadvantaged janajati	1177 (28.3)	4143 (71.7)	0.9 (0.8,0.9)**
Disadvantaged non-dalit Terai caste	198 (17.9)	916 (82.1)	0.5 (0.5,0.6)**
Religious minorities	19 (18.9)	72 (81.1)	0.5 (0.4,0.6)**
Relatively advantaged janajati	242 (21.3)	1003 (78.7)	0.6 (0.5,0.6)**
Upper caste	1418 (32.1)	3588 (67.9)	1.0
Pregnancy			
No	3011 (26.1)	10480 (73.9)	0.2 (0.2,0.3)**
Yes	406 (62.4)	325 (37.6)	1.0
Ecological regions			
Mountain	20 (2.2)	881 (97.8)	0.05 (0.04,0.07)**
Hill	2165 (30.2)	6167 (69.8)	1.9 (0.9,1.0)
Terai	1232 (27.6)	3757 (72.4)	1.0

^a^Weighted percent

aOR^b^ weighted adjusted odds ratio, 1 = reference

^c^The odds ratio are adjusted for all variables

**P < 0.001

Janajati includes all the castes that fall under marginalized group except dalit (lower caste)

## Discussion

This study showed that nearly a third of women in Terai were malnourished and on average, the malnourished women were 16.4 % in Nepal. This is almost similar to the national data [3] and lower than that of Bangladesh (24.2 %) [14] and India, where the malnourished women, as indicated by low BMI, were 35.6 % in 2006 [15]. There has been decrement in malnourishment in women in Nepal due to interventions on maternal health, nutrition and other women empowering programs through programs such as School Health and Nutrition (SHN), *Suaahara*, multisectoral nutrition plan (MSNP), Knowledge-based Integrated Sustainable Agriculture and Nutrition (KISAN) and Agriculture and Food Security Project (AFSP) launched by government and non-government organizations [16–18]. Despite this progress, the current rate of under-nutrition remains in unacceptable condition. When we compared BMI among the ecological regions, there was a big difference in the prevalence of malnourished women; Terai being the most affected one. The reason might be poor nutrition and maternal health in the region. Therefore, it would be better if the government of Nepal intensify nutrition and education programs to improve the nutritional status.

In Terai, about one-fifth of the women did not possess land for kitchen garden. With the possession of kitchen garden, women are likely to be involved in agriculture by growing vegetables and fruits that can ultimately help in the reduction of nutrients deficiency [19, 20]. In addition, education and awareness are essential to ensuring that expanded and more diverse production translates into healthier diets and better nutrition [20]. In the present study, about 95 % of women used iodized salt, which, according to WHO, showed that Nepal’s salt iodization program is considered to be on a good track to eliminate iodine deficiency. In Bangladesh, 82.3 % of the households have adequately iodized salt [14], while another report in Nepal shows that about three-fourth of households have adequately iodized salt [3]. Iodine deficiency is related to adverse pregnancy outcomes such as abortion, fetal brain damage and congenital malformation, stillbirth, and perinatal death [21]. Therefore, use of iodized salt by women of reproductive age is essential. The majority of women in all ecological regions consumed junk foods. As such foods do not provide adequate nutrition, they should be discouraged and food system strategies can be adopted to prevent micronutrient malnutrition [22].

In our study, in Terai, the majority of women in their households produced cereals. However, comparatively fewer women produced vegetables there. This might be because cereals are staple foods and can be sold later on, whereas, vegetables cannot be stored for a longer time to sell. Production of fruits was the lowest than other products in all ecological regions. Low production of vegetables and fruits can be attributed to micronutrient deficiency disorders as agricultural production has an impact on nutrition [19, 20]. If women of reproductive age do not consume proper diet including enough fruits and vegetables, it is likely that they suffer from different micronutrients deficiencies [16]. Irrespective of agricultural production, it is very essential for them to consume such foods daily at proper intervals.

This study revealed that majority of women in Nepal depends upon cereals to fulfill their energy need. These foods have become the sole source of energy. The findings are in concordance with a study done in Bangladesh and Mozambique, where dietary patterns are heavily dominated by starchy staples [23]. Pulses/legumes are rich source of plant proteins and have many health benefits [24]. The women can fulfill the daily need of proteins from local products like pulses/legumes that can be grown in their own fields. However, more women consumed pulses/legumes once a week in the Mountain region. A large sample study in India shows that 87.8 % of married women consumed pulses or beans once a week [10]. This suggests that the women are less likely to get enough protein content. When there is scarcity of carbohydrates and proteins in the diet, the women might suffer from protein-energy malnutrition. Consequently, there can be low birth weight children, decreased mental and physical ability in children, still birth and even maternal death [25].

Micronutrients can be obtained from vegetables, fruits, meat and milk/milk products and are essential for women especially during pregnancy and lactation [26]. The higher the consumption of these foods, the less likely is suffering from micronutrients deficiency. The present study revealed that the frequency of consumption of vegetables was lower in Terai. This can be related to the fact that majority of women in Terai are anemic [27] as they supposedly do not eat dark green leafy vegetables rich in iron. The majority of women in all the ecological regions consumed meat and fruits once a week. In India, the frequency of consumption of meat and fruits was once a week in 31.9 and 33.0 % of women [10]. Nearly three out of ten women consumed milk and milk products at least once a day. The low frequency of consumption of items from food groups such as meat and fruits could be due to the condition that only a few households could afford to buy such foods on a daily basis. In addition, despite the affordability, sometimes it may be difficult to access such foods due to either a lack of production in certain regions (geographical constraints) or certain intra-household decisions on food consumption. And importantly, there might be either a lack of awareness about balanced nutritional intake or specific food preferences. According to a report, women consuming foods from five or more food groups out of ten have a greater likelihood of meeting their micronutrient needs than women consuming foods from fewer food groups [28]. The consumption of such foods rich in micronutrients should be encouraged in order to prevent micronutrient deficiencies and ensure safe women’s health [25]. In addition, agricultural interventions for the production and consumption of such foods to improve nutrition [29] are imperative in Nepal.

In the present study, surprisingly, the women who had formal education were more likely to get malnourished than the women having informal education. This needs to be verified from other large-scale studies. Further, although women had formal education, the majority of them (48.5 %) were unemployed (results not shown). Consequently, it results in poor nutrition. Employment of women benefit to household nutrition in general and the woman’s nutritional status in particular [30]. In addition, higher education, especially for women of reproductive age in Nepal does not necessarily indicate better nutrition education; hence, they could be malnourished. This is supported by our findings from Table 5: those women who did not get nutrition education from any organizations were more likely to get malnourished. This finding is supported by a study done in Pakistan [31]. This suggests that the educated women should also be kept in priority while formulating the nutrition policies and implementing them. The younger the women are the more there is chance of being malnourished because of immature physiological conditions and poor knowledge in nutrition at that age. In addition, there is increased demand of nutrients in the body during this age span [32]. Our study showed that unemployed women had more chances of being malnourished than women doing manual work. This finding is supported by a study done in Ethiopia [30]. In Nepal, ethnicity played an important role in determining the nutritional status of women. Although women of upper caste were more likely to be malnourished than the women of religious minorities, higher percentage of women from disadvantaged non-dalit Terai caste are malnourished, and focus should be given to such ethnic groups. Studies have shown that social determinants like disadvantaged groups, ethnic and regional disparities play a significant role in under-nutrition [33–35]. In addition, the ecological regions affect the nutritional status of women in Nepal. Despite the fertile soil in Terai, more women here were malnourished. This could be because of lack of nutrition education provided to them [31, 36]. Therefore, the government and non-government organizations should work together to bring down the higher rate of under-nutrition in women, especially in Terai region.

Anemia can result from different conditions, but the major cause of anemia is due to iron deficiency [37]. In the present study, deviating from common belief, the women having formal education were more likely to be anemic than women having informal education and illiterate women. This contrasts a study done among pregnant women in India [38] and Pakistan [39]. Therefore, educated women should also be included under the surveillance while combating with micronutrient deficiency and special policies are needed to be formulated to address such situation. Similarly, the women who were unemployed were more likely to be anemic that the women doing manual work. This could be due to reason that employment generates income and women are likely to consume iron rich foods [40]. However, in a study done among pregnant women in Pakistan, the employed women were more anemic than unemployed ones [39]. Although women were employed in Pakistan, they were underpaid or had lower income, which ultimately resulted in lower consumption of iron rich foods. Surprisingly, the women who were involved in agriculture were also anemic. This underscores the importance of knowledge of cultivation of foods that are rich in nutrition and proper feeding habits of women. Almost all of the ethnic group women were anemic, the women of upper caste and dalit being more anemic. A study conducted in Vietnam highlights the multi-causal etiology of anemia including ethnicity and socio-economic status in women of reproductive age [41]. The condition was even worse in the women living in Terai. The women in Terai were more likely to be anemic than the women in Mountain. The negative effects of anemia can be premature birth, low birth weight, infant mortality and maternal morbidity and mortality [42]. The present study revealed that the pregnant women were more likely to be anemic than the normal ones. Several studies have shown higher rates of anemia in pregnant women ranging from 56.8 % in urban eastern Ethiopia [43], 89.6 % in northern India [44] to 90.5 % in urban Pakistan [39]. This could be due to high iron demand during pregnancy [45]. Iron deficiency anemia during pregnancy is harmful to both mother and child [46]. The government of Nepal recommends additional supplements like iron/folate after first trimester only. However, women need nutritious food with supplements throughout their reproductive age in order to be healthy and have healthy babies.

## Conclusions

The dietary intake patterns to combat against nutritional deficiencies are not appropriate and nutritional status of women of reproductive age is still poor. The majority of women consume starchy staple food while less attention has been given to the consumption of vegetables, meat, fruits and dairy products. Cultivation of vegetables and fruits and consuming them can prove to be an important factor in maintaining better nutritional status. It is imperative that the government and non-government organizations act to improve dietary intake pattern of women in Nepal to promote women’s health.

## Abbreviations

aOR: Adjusted Odds Ratio
BMI: Body Mass Index
CI: Confidence Interval
FCHVs: Female Community Health Volunteers
NHRC: Nepal Health Research Council
SPSS: Statistical Package for Social Sciences
VDC: Village Development Committee
WHO: World Health Organization

## References

[1] Togo P, Osler M, Sorensen TI, Heitmann BL (2001). Food intake patterns and body mass index in observational studies. Int J Obes Relat Metab Disord.

[2] Osler M, Heitmann BL, Hoidrup S, Jorgensen LM, Schroll M (2001). Food intake patterns, self rated health and mortality in Danish men and women. A prospective observational study. J Epidemiol Community Health.

[3] Ministry of Health and Population (MOHP), New ERA, ICF International Inc (2012). National Demographic and Health Survey, 2011.

[4] Black RE, Allen LH, Bhutta ZA, Caulfield LE, de Onis M, Ezzati M (2008). Maternal and child undernutrition: global and regional exposures and health consequences. Lancet.

[5] Ransom IE, Elder KL (2003). Nutrition of women and adolescent girls: why it matters?.

[6] Huffman LS, Baker J, Shumann J, Zehner RE (1999). The case for promoting multiple vitamin and mineral supplements for women of reproductive age in developing countries. Food Nutr Bull.

[7] Demissie T, Mekonen Y, Haider J (2003). Agro-ecological comparison levels and correlate of nutritional status of women. Ethiop J Health Dev.

[8] Branca F, Piwoz E, Schultink W, Sullivan LM. Nutrition and health in women, children, and adolescent girls. BMJ. 2015;351. 10.1136/bmj.h4173 26371218

[9] Central Bureau of Statistics (2012). National population and housing census 2011.

[10] Padmadas SS, Dias JG, Willekens FJ (2006). Disentangling women’s responses on complex dietary intake patterns from an Indian cross-sectional survey: a latent class analysis. Public Health Nutr.

[11] Bruce C (2001). Anthropometric indicators measurement guide.

[12] World Health Organization (2006). BMI classification.

[13] Chernecky CC, Berger BJ (2001). Laboratory tests and diagnostic procedures.

[14] National Institute of Population Research and Training (NIPORT), Mitra and Associates, ICF International (2013). Bangladesh demographic and health survey 2011.

[15] International Institute for Population Sciences (IIPS), Macro International (2007). National Family Healthy Survey (NFHS-3 ), 2005–2006: India.

[16] Bhandari S, Banjara MR (2015). Micronutrients deficiency, a hidden hunger in Nepal: prevalence, causes, consequences, and solutions. Int Scholarly Res Notices.

[17] Houston R, Shrestha MB, Pomeroy A, Wun J, Sharma I (2014). Pathways to better nutrition case study: Nepal strategic background report.

[18] Ministry of Health and Population Nepal, Partnership for Maternal NCH, WHO, World Bank, Alliance for Health Policy and Systems Research (2014). Success factors for women’s and children’s health: Nepal.

[19] Ruel MT, Alderman H, Maternal and Child Nutrition Study Group (2013). Nutrition-sensitive interventions and programmes: how can they help to accelerate progress in improving maternal and child nutrition?. Lancet.

[20] International Fund for Agricultural Development (IFAD) (2014). Improving nutrition through agriculture.

[21] Zimmermann MB (2012). The effects of iodine deficiency in pregnancy and infancy. Paediatr Perinat Epidemiol.

[22] Miller DD, Welch RM (2013). Food system strategies for preventing micronutrient malnutrition. Food Policy.

[23] Arimond M, Wiesmann D, Becquey E, Carriquiry A, Daniels M, Deitchler M (2011). Dietary diversity as a measure of the micronutrient adequacy of women’s diets in resource-poor areas: summary of results from five sites.

[24] Ofuya Z, Akhidue V (2005). The role of pulses in human nutrition: a review. J Appl Sci Environ Manag.

[25] Black RE, Victora CG, Walker SP, Bhutta ZA, Christian P, de Onis M (2013). Maternal and child undernutrition and overweight in low-income and middle-income countries. Lancet.

[26] Zerfu TA, Ayele HT (2013). Micronutrients and pregnancy; effect of supplementation on pregnancy and pregnancy outcomes: a systematic review. Nutr J.

[27] Bhandari S, Sayami JT, Sayami M, Kandel BP, Banjara MR (2014). General health status of women of reproductive age in Nepal. J Nepal Health Res Counc.

[28] Introducing the minimum dietary diversity–women (MDD-W): global dietary diversity indicator for women, July 15–16. Washington, DC. 2014. http://www.fantaproject.org/sites/default/files/resources/Introduce-MDD-W-indicator-brief-Sep2014.pdf. Accessed 02 Nov 2015.

[29] World Bank (2007). From agriculture to nutrition. Pathways, synergies and outcomes.

[30] Girma W, Genebo T (2002). Determinants of nutritional status of women and children in Ethiopia Calverton.

[31] Zahid Khan A, Rafique G, Qureshi H, Halai BS (2013). A nutrition education intervention to combat undernutrition: experience from a developing country. ISRN Nutr.

[32] Story M, Hermanson J, Story M, Stang J (2000). Nutrient needs during adolescence and pregnancy. Nutrition and the pregnant adolescent: a practical reference guide.

[33] Kavosi E, Hassanzadeh Rostami Z, Kavosi Z, Nasihatkon A, Moghadami M, Heidari M (2014). Prevalence and determinants of under-nutrition among children under six: a cross-sectional survey in Fars province, Iran. Int J Health Policy Manag.

[34] Brcanski J, Jovic-Vranes A, Marinkovic J, Favre D (2014). Social determinants of malnutrition among Serbian children aged <5 years: ethnic and regional disparities. Int J Public Health.

[35] Jesmin A, Yamamoto SS, Malik AA, Haque MA (2011). Prevalence and determinants of chronic malnutrition among preschool children: a cross-sectional study in Dhaka city, Bangladesh. J Health Popul Nutr.

[36] Bhutta ZA, Ahmed T, Black RE, Cousens S, Dewey K, Giugliani E (2008). What works? Interventions for maternal and child undernutrition and survival. Lancet.

[37] Miller JL (2013). Iron deficiency anemia: a common and curable disease. Cold Spring Harb Perspect Med.

[38] Lokare P, Karanjekar V, Gattani P, Kulkarni A (2012). A study of prevalence of anemia and sociodemographic factors associated with anemia among pregnant women in Aurangabad city, India. Ann Niger Med.

[39] Baig-Ansari N, Badruddin SH, Karmaliani R, Harris H, Jehan I, Pasha O (2008). Anemia prevalence and risk factors in pregnant women in an urban area of Pakistan. Food Nutr Bull.

[40] Kim JY, Shin S, Han K, Lee KC, Kim JH, Choi YS (2014). Relationship between socioeconomic status and anemia prevalence in adolescent girls based on the fourth and fifth Korea National Health and Nutrition Examination Surveys. Eur J Clin Nutr.

[41] Nguyen PH, Gonzalez-Casanova I, Nguyen H, Pham H, Truong TV, Nguyen S (2015). Multicausal etiology of anemia among women of reproductive age in Vietnam. Eur J Clin Nutr.

[42] Allen LH (2000). Anemia and iron deficiency: effects on pregnancy outcome. Am J Clin Nutr.

[43] Addis Alene K, Mohamed DA (2014). Prevalence of anemia and associated factors among pregnant women in an urban area of eastern Ethiopia. Anemia.

[44] Bora R, Sable C, Wolfson J, Boro K, Rao R (2014). Prevalence of anemia in pregnant women and its effect on neonatal outcomes in Northeast India. J Matern Fetal Neonatal Med.

[45] Khalafallah AA, Dennis AE (2012). Iron deficiency anaemia in pregnancy and postpartum: pathophysiology and effect of oral versus intravenous iron therapy. J Pregnancy.

[46] Coad J, Conlon C (2011). Iron deficiency in women: assessment, causes and consequences. Curr Opin Clin Nutr Metab Care.

